# Survey dataset on occupational hazards on construction sites

**DOI:** 10.1016/j.dib.2018.04.028

**Published:** 2018-04-13

**Authors:** Patience F. Tunji-Olayeni, Adedeji O. Afolabi, Obiora I. Okpalamoka

**Affiliations:** Covenant University, Nigeria

**Keywords:** Accidents, Construction industry, Craftsmen, Health, Occupational hazards

## Abstract

The construction site provides an unfriendly working conditions, exposing workers to one of the harshest environments at a workplace. In this dataset, a structured questionnaire was design directed to thirty-five (35) craftsmen selected through a purposive sampling technique on various construction sites in one of the most populous cities in sub-Saharan Africa. The set of descriptive statistics is presented with tables, stacked bar chats and pie charts. Common occupational health conditions affecting the cardiovascular, respiratory and musculoskeletal systems of craftsmen on construction sites were identified. The effects of occupational health hazards on craftsmen and on construction project performance can be determined when the data is analyzed. Moreover, contractors’ commitment to occupational health and safety (OHS) can be obtained from the analysis of the survey data.

**Specifications Table**

**Table:** 

Subject area	*Construction*
More specific subject area	*Occupational Health Hazards*
Type of data	*Tables, figures and text files*
How data was acquired	*Field Survey*
Data format	*Raw*
Experimental factors	*Purposive sampling of construction craftsmen comprising of bricklayers, carpenters, painters, plumbers, welders, electricians and steel fixers.*
Experimental features	*Descriptive statistics of the occupational health conditions of craftsmen, effects of occupational health hazards on construction project performance and contractors’ commitment to occupational health and safety are presented.*
Data source location	*Mainland Area of Lagos, Lagos State, Nigeria*
Data accessibility	*The data are in this data article*

**Value of the data**

• The dataset provided showed the empirical evidence of health challenges in the cardiovascular and respiratory systems including the musculoskeletal disorders experienced by craftsmen on construction sites.
• The understanding of the dataset sheds light on the effect of occupational health hazards on the well-being of the craftsmen and construction project performance.
• The dataset gives insight into the level of contractors’ commitment to occupational health and safety (OHS).
• Construction activities and materials are crucial entities that can impact the body system when not handled in the standardized way, therefore, the dataset can guide policies on reducing occupational health hazards among craftsmen on construction sites.
• The structured questionnaire can be adopted or modified for similar research in a larger context in sub-Saharan Africa and other parts of the world. In addition, comparison can be drawn with other sectors where workers are more prone to be affected by working conditions and severe exposure to toxic materials.

## 1 Data

**Fig. 1:**
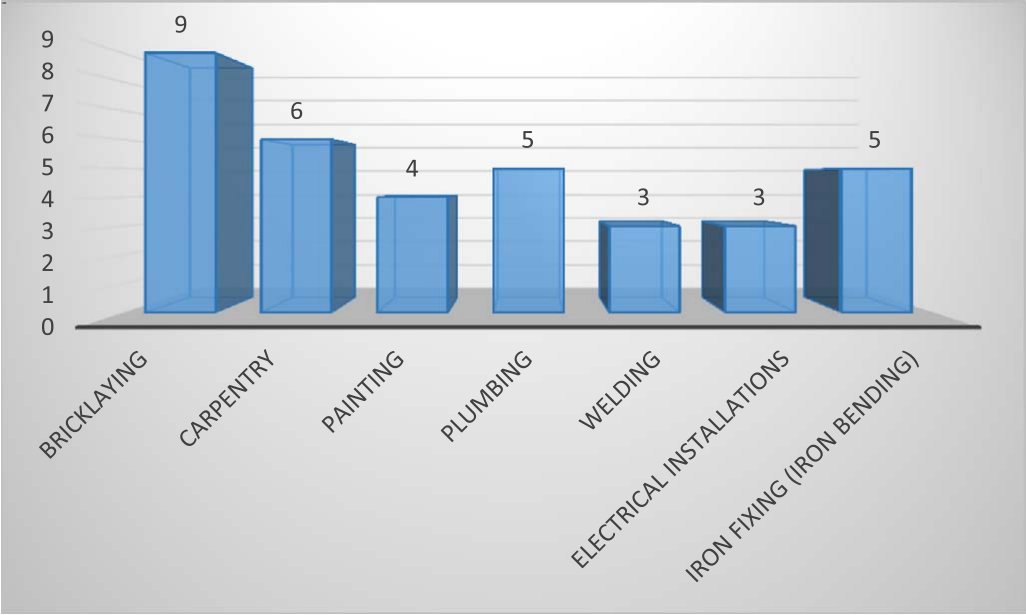
Overview of craftsmen surveyed.

**Fig. 2:**
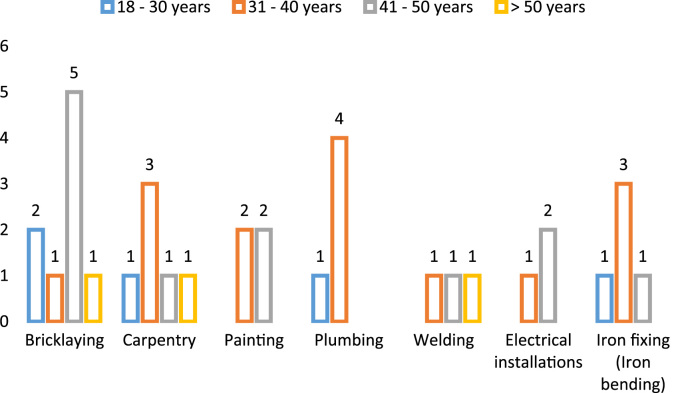
Summary of age category of the craftsmen.

**Fig. 3:**
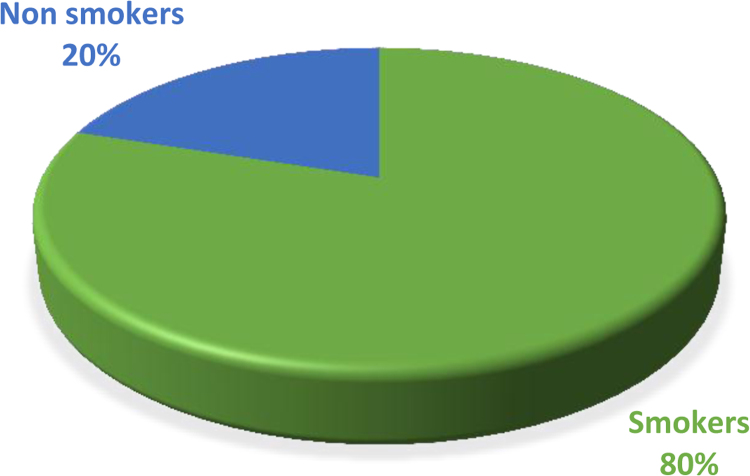
Breakdown of smoking and non-smoking craftsmen.

**Fig. 4:**
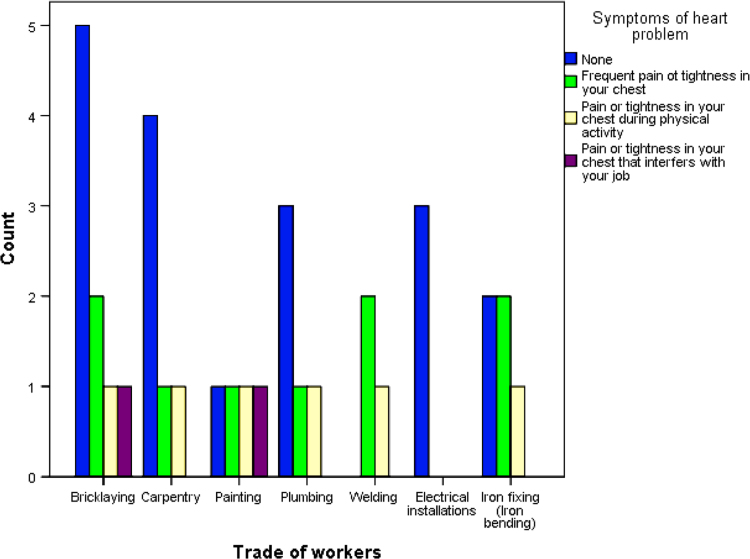
Heart challenges among craftsmen.

**Fig. 5:**
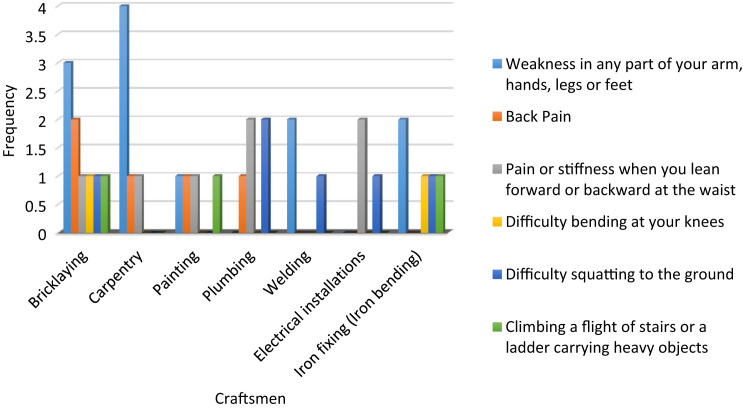
Musculoskeletal Disorders (MSDs) among craftsmen.

**Fig. 6:**
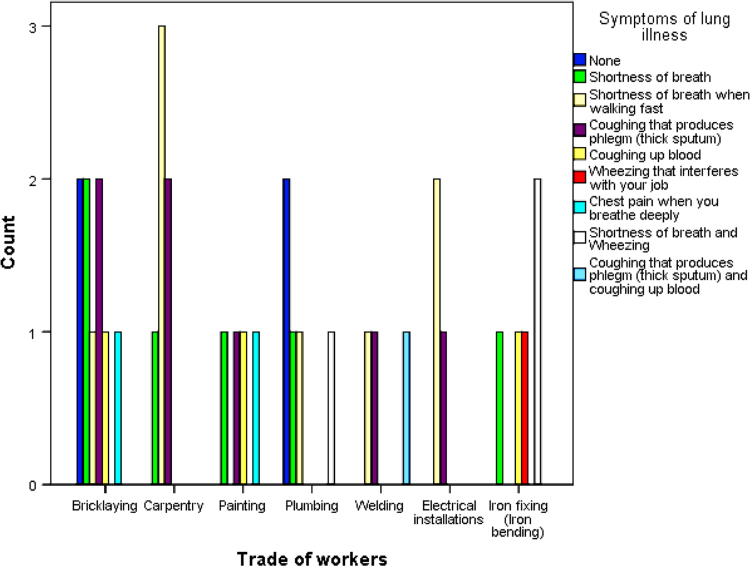
Symptoms of lung illness among craftsmen.

**Fig. 7:**
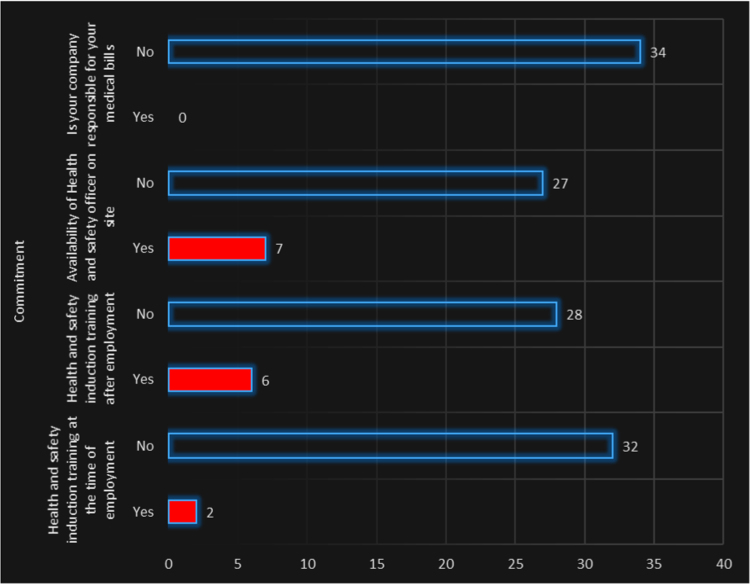
Contractors’ commitment to occupational health and safety.

The cardiovascular, respiratory and musculoskeletal systems are essential parts of the human body that could be severely affected by the un-friendly working conditions of construction sites and exposure to some chemical and toxic building materials [1], [2], [3], [4]. The dataset presented are responses obtained from the distribution of questionnaires to thirty-four (34) craftsmen working on different construction sites in Lagos Mainland Area of Lagos State, Nigeria. The questionnaire was designed to elicit information on occupational health hazards on construction sites. Craftsmen were selected in the dataset because they one of the most influential in erecting building and civil structures on construction sites. Fig. 1 shows the breakdown of the nature of craftsmen in the dataset. The participants’ information showed there were steel fixers (5), electricians (3), welders (3), plumbers (5), painters (4), carpenters (6) and bricklayers (9). Fig. 2 presented the age category of the craftsmen, 5 of the craftsmen were between 18 and 30 years, while 15 of them were between 31 and 40 years, 12 craftsmen were between 41 and 50 years and 3 of the craftsmen were above 50 years. From Fig. 3, it can be observed that 80 percent of the craftsmen smoked while only 20 percent were non-smokers. Fig. 4 showed the summary of craftsmen with heart challenges. From Fig. 4, it can be observed that 9 craftsmen stated that they have frequent pain or tightness in the chest, 6 complained of pain or tightness in your chest during physical activity while only 2 craftsmen complained that the pain or tightness in their chest interfered with their jobs. Fig. 5 presented the Musculoskeletal Disorders (MSDs) among craftsmen. A breakdown of the musculoskeletal symptoms experienced by the craftsmen are weakness in any part of the arm, hands, legs or feet (12), back pain (5), pain or stiffness when you lean forward or backward at the waist (7), difficulty bending the knees (2), difficulty squatting to the ground (6) and climbing a flight of stairs or a ladder carrying heavy objects (3). The symptoms of respiratory challenges experienced by the craftsmen in Fig. 6 are shortness of breath (6), shortness of breath when walking fast (8), coughing that produces phlegm (thick sputum) (7), coughing up blood (3), wheezing that interferes with your job (1) chest pain when you breathe deeply (2), shortness of breath and wheezing (3) and coughing that produces phlegm (thick sputum) and coughing up blood (1). Fig. 7 highlighted contractors’ commitment to occupational health and safety such as Health and safety induction training at the time of employment, health and safety induction training after employment, availability of Health and safety officer on site and responsibility for your medical bills. Table 1 showed some effect of occupational health hazard on construction project performance such as reduces worker's productivity, threatens the livelihood of construction workers, drains the income of workers, results in poor work environment, absenteeism, results in workers' dissatisfaction, causes loss of skilled/ experienced workers, causes disability, leads to illness and leads to loss of life. Further analysis of the data can provide inferential decisions about health conditions of craftsmen in relation to their trade and the commitment or provisions by the contractors to the wellbeing of the craftsmen. The dataset is attached as supplementary data 1.

**Table 1:** Effects of occupational health hazards.

**Effect**	**Min.**	**Max.**	**Mean score**	**Std. deviation**	**Ranking index**
Reduces worker's productivity	4	5	4.97	.169	1st
Threatens the livelihood of construction workers	4	5	4.74	.443	2nd
Drains the income of workers	3	5	4.69	.583	3rd
Results in poor work environment	3	5	4.43	.655	4th
Absenteeism	3	5	4.09	.507	5th
Results in workers' dissatisfaction	2	5	4.00	.542	6th
Causes loss of skilled/ experienced workers	3	5	3.94	.639	7th
Causes disability	2	5	3.54	.741	8th
Leads to illness	2	5	3.43	.979	9th
Leads to loss of life	2	5	3.37	1.031	10th

## 2 Experimental design, materials and methods

This dataset is based on previous research conducted on occupational health hazards of craftsmen on construction sites [1], [2], [3], [4]. The population comprised of craftsmen working in the Nigerian construction industry. The respondents comprised of craftsmen in the construction industry who are bricklayers, carpenters, painters, plumbers, welders, electricians and steel fixers. Survey was used because it can provide understanding and predictions into respondents’ characteristics. Using a purposive sampling technique due to the characteristics of the respondents, a total of one hundred (100) questionnaires were distributed to craftsmen working on different construction sites in Lagos Mainland Area of Lagos State, Nigeria. Out of which thirty five (35) questionnaires were returned, representing 35% response rate. The dataset area was selected due to the high number of ongoing and completed construction projects located within the vicinity. The data instrument was a closed ended questionnaire which obtained participants’ information of trade and age. Other questions in the instrument were based on the health conditions of the craftsmen, effect of their health condition on their work performance and the state of contractors’ commitment to their well-being while performing their duties. The dataset obtained was analyzed using SPSS and Microsoft excel. Details of similar research that have analyzed dataset using descriptive statistics can be found in [5], [6], [7], [8], [9], [10], [11], [12], [13], [14], [15], [16], [17], [18], [19], [20], [21].
